# Initiating a Fetal Cardiac Program from Scratch in Low- and Middle-Income Countries: Structure, Challenges, and Hopes for Solutions

**DOI:** 10.1007/s00246-024-03479-9

**Published:** 2024-04-19

**Authors:** Shaimaa Rakha

**Affiliations:** Pediatric Cardiology Unit, Department of Pediatrics, Faculty of Medicine, Mansoura University, Mansoura, Egypt

**Keywords:** Fetal echocardiography, Program, Low- and middle-income countries, Challenges

## Abstract

Although fetal cardiac programs are well established in developed countries, establishing an efficient program in low- and middle-income countries (LMICs) is still considered a significant challenge. Substantial obstacles usually face the initiation of fetal cardiac service from scratch in LMICs. The primary structural frame of a successful fetal cardiac program is described in detail, emphasizing the required team members. The potential challenges for starting fetal cardiac services in LMICs include financial, awareness-related, prenatal obstetric screening, sociocultural, psychosocial, and social support factors. These challenges could be solved by addressing these barriers, such as collecting funds for financial support, raising awareness among families and health care providers, telemedicine, building international health partnerships, modifying training protocols for fetal cardiologists and sonographers, and initiating support groups and social services for families with confirmed fetal cardiac disease. Initiating a successful fetal cardiac program requires multi-aspect structural planning. The challenges for program initiation require diverse efforts, from modified training and promoting awareness of care providers and the community to governmental and nonprofit organizations’ collaborations for proper building and utilization of program resources.

## Introduction

The fetus is considered the new patient of the century with utmost care and development in the antenatal medical field. Since 1972, when Winsberg was the first to report fetal M-mode echocardiography, fetal cardiology has undergone tremendous advancements since the original description nearly 50 years ago [1]. Due to the improvements in fetal cardiac diagnostic techniques, antenatal cardiac service is a well-established form of health care in developed high-income countries [2, 3]. Moreover, antenatal fetal cardiac interventions are successfully applied to manage some critical congenital heart diseases (CHDs) in selected centers [4–6].

On the other side, a large share of infant mortality in low- and middle-income countries (LMICs) is still attributed to the delay in recognition of critical CHDs [7, 8]. As a limited percentage of cases are diagnosed before birth in these countries, critical CHD cases are often unrecognized and usually succumb due to their lesion in the neonatal period or early infancy [9]. The late diagnosis in LMICs is mainly related to the limited availability and access to fetal cardiac health services. Although it is mandatory to initiate and expand, establishing an efficient fetal cardiac program in LMIC is still a significant challenge.

In the upcoming sections, light will be shed on the primary rationale for establishing a fetal cardiac program. A structural framework will be suggested for such a program, along with exploring challenges and obstacles in LMIC and discussing possible hopes for solutions.

### The Rationale of Fetal Heart Program in LMICs

CHD is one of the most common forms of congenital anomalies; the birth prevalence of CHD increased to a maximum in the period of 2010–2017 to reach 9.41/1000 [10]. From 1990 to 2017, the rank of CHD increased from ninth to seventh globally and from 17 to 11th in low-socio-demographic index (SDI) countries, and improvement in CHD mortality lagged behind other mortality causes. Of the 261.247 deaths caused by CHD globally in 2017, 180.624 were among infants in countries on the low- and low-middle SDI quintiles [11].

Fetal cardiac disorders could be diagnosed in antenatal life with high sensitivity and specificity [12, 13]. Defects of the heart can be detected as early as 16–18 weeks gestation using transabdominal fetal echocardiography and earlier in the first trimester with a transvaginal approach. The list of indications for detailed fetal echocardiography described by the American Society of Echocardiography (ASE) warrants the need for specialized fetal cardiac services [14].

It is now well established that fetal echocardiography has a wide range of perinatal benefits regarding management and outcomes. Prenatal diagnosis significantly reduces poor general conditions on admission and improves survival and surgical outcomes in babies with antenatally diagnosed CHD [15, 16]. Prenatal diagnosis, particularly prior to 24 weeks of gestation, allows valuable time to consider options, making informed choices and plans for how to proceed according to the diagnosed CHD. Moreover, fetal diagnosis with planned peripartum care in low-resource settings was found to be associated with significantly lower costs of neonatal cardiac care and improved preoperative status in resource-limited settings, especially for critical CHD [17, 18].

## Structure of the Program

**Fig. 1:**
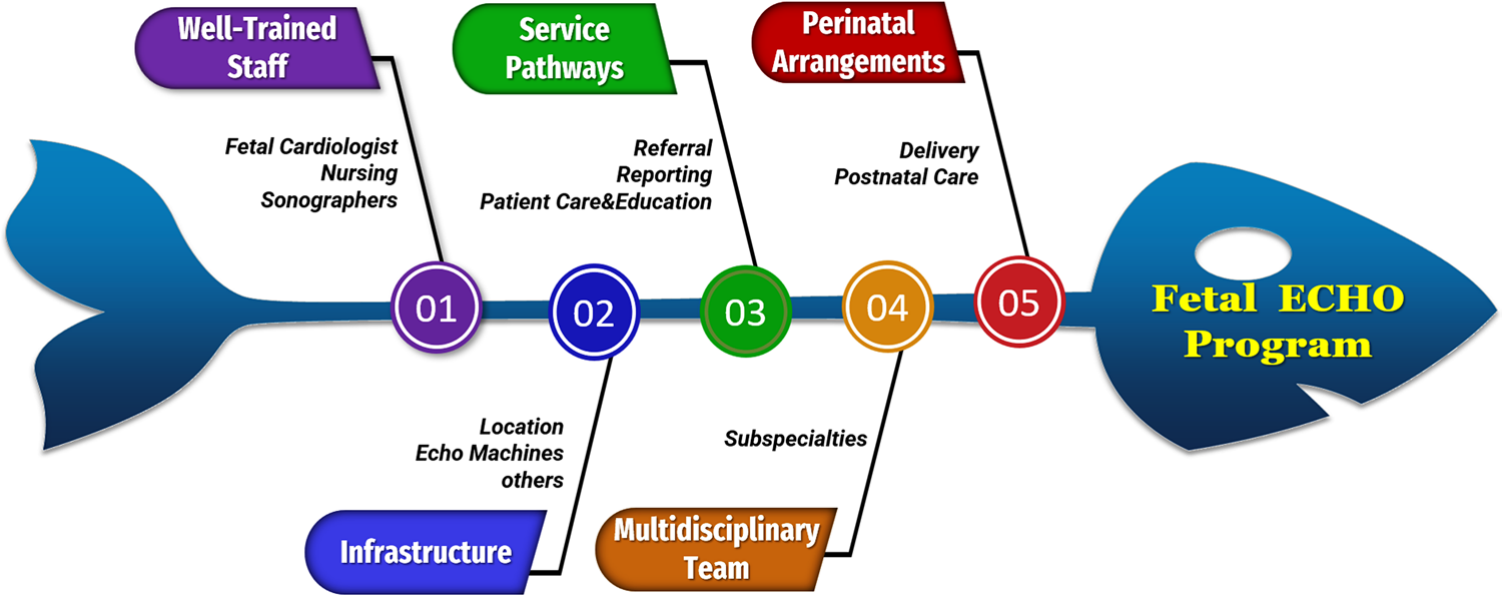
Fishbone diagram illustrating the structure of a successful fetal echocardiography program

The current review suggests a five-element structure for initiating an efficient and successful fetal cardiac program. The suggested structure will be discussed in detail in the next section. A diagram of the structural framework is demonstrated in Fig. 1.

### Well-Trained Fetal Cardiac Staff

A fetal heart-oriented team is the basis of the service to have a strong-based structure for the fetal cardiac program. It should include well-trained fetal cardiologists, nurse coordinators, or advanced practice nurses with the possibility of additional help from sonographers and secretary service [19].

#### Fetal Cardiologists

Experienced, well-trained fetal cardiologists are the cornerstone of a successful fetal echocardiography program. They provide accurate diagnoses, which is the base for arranging management plans, including surveillance, antenatal medication, or intervention, and provide familial counseling for available options, expected survival, expectations, and associations. In addition, they have an educational role for other team members, including sonographers, nurses, and obstetricians [20].

#### Nurse Coordinator or Advanced Practice Nurse

The nurse is the first orientation source of the referred cases regarding fetal echocardiography. A brief introduction can be offered at the initial visit with information regarding the safety of fetal echocardiograms, study duration, possible limitations, and potential follow-up visits. After diagnosis, the nurse helps in counseling parents about educational materials and guides support groups after the initial session with the fetal cardiologist, ongoing support to families, arranges follow-up appointments and referrals, and contacts the pregnant lady’s local hospital [19].

#### Fetal Cardiac Sonographers/Specialist Radiographers

In some countries, a sonographer or radiographer can perform fetal cardiac scans under the supervision of a consultant trained in fetal cardiology. However, fetal cardiac-trained sonographers are limited or are not available at all in some of the developing countries such as Egypt.

#### Secretary

The presence of a secretary on the fetal heart team is additive to the service for arranging appointments, data entry, and sometimes performing fundamental statistical analysis regarding the program.

### Infrastructure

Essential infrastructural items should be available to start a fetal echocardiography program. These requirements include the basic physical and organizational needs to start the service process. The key infrastructural required components include the following.

#### Echocardiography Machine

Echocardiographic machine with fetal echocardiographic software supported with capabilities for performing basic echocardiographic modalities such as (2D, Color Flow, Pulsed Doppler, M.mode) is mandatory for program initiation. Advanced modalities like tissue Doppler, Spatio‐temporal image correlation (STIC), 3D echocardiography, and Speckle tracking could add to the diagnostic yield  of fetal echocardiography when available. The echocardiographic machine should be equipped with a high-frequency probe with optimum resolution to delineate details, especially curvilinear probes, which are more patient-friendly for transabdominal echocardiograms. A transvaginal probe could be considered if the service is expanded to the first trimester fetal echocardiography.

#### Location

The fetal echocardiography room or clinic should include an adult-sized bed with sufficient privacy for the pregnant lady. A quiet place to break the bad news is preferred. The room should include diagrams of normal and abnormal hearts and fetal circulation to aid in parental understanding of the problem [20]. However, some fetal cardiologists have the skill to draw simplified demonstrative diagrams for the parents. The location of the fetal echocardiography room is preferred to have physical proximity to other services such as fetal medicine, obstetrics, neonatology unit, and pediatric cardiology.

#### Storage of Echocardiographic Studies

A record should be kept of all performed fetal echocardiographic studies. Old echocardiographic machines allowed videotaping. Nowadays, digital recordings of all scans are for further review and analysis. Some hospitals have online storage on PACs for DICOM form of echocardiographic studies, with the possibility of uploading the studies to an online cloud with a system to obtain and record them. This allows for integration with computerized reporting software and databases for data entry of all cases to allow auditing, research, and development. Whenever cloud storage is unavailable, which is a frequent limitation in LMIC, fetal echocardiographic studies should be stored on hard drives. However, this has several disadvantages, such as possible loss or unrecorded data and the limited storage capacity compared to cloud storage.

### Designing Service Pathways

#### Referral System

Establishing an efficient referral system for fetal cardiac service should be clearly defined by the fetal cardiologist to the involved teams. This should include clarification of the accepted lowest gestational age for the service and the indications of fetal echocardiography as recommended by ASE [14]. It is essential to formulate a fetal echocardiogram request form to be filled in by the referring physician to accurately indicate the referral reason and the priority of the referral, as some referrals could be seen on an elective basis. On the contrary, other disorders, such as fetal arrhythmias and hydrops fetalis, should be assessed on an urgent basis.

For initiated programs, awareness building about the program for referring physicians is the most critical initiative, as the lack of awareness and late referrals cause low utilization of services [8]. Earlier studies showed that the major indication for fetal echocardiography was a family history of CHD [21, 22]. However, studies in the twenty-first century demonstrated the rising role and yield of referrals due to suspected abnormality on obstetric screens [23–25]. Hence, the education and orientation of the obstetric team are of utmost importance for service utilization and progression.

#### Reporting System

Efficient fetal echocardiograms reporting is required to accurately document the cardiac diagnosis’s details and the care plan to guide the other managing team members inside or outside the hospital. The report should be available on the hospital information system, preferably linked to the echocardiographic stored studies. Email-sent copies are always used; however, printed paper copies are required in LMICs, primarily when the patient will be referred to an outreach service or a primary physician.

#### Patient Care and Education Pathway

A designed pathway should be clearly defined for utmost care. For the initial visit to the fetal echocardiography laboratory, the first one of the team to meet the patient and describe the pathway of the service should be determined. The preliminary information regarding service pathway should be described to the patient, such as the expected study duration, safety,  and who will perform the fetal echocardiography; the consultant, the fellow, or the sonographer if available. In addition, the next step should be flagged which is interpretation of the fetal echocardiogram and consultation process with the fetal cardiologist.

For the post-diagnosis process, the counseling process should delineate the required information for the parents, such as lesion description, available management options, arrangements for follow-up care such as next appointment, required consultations, expected effect on delivery time, delivery options, and finally, offering brochures and parent information sheets, including links to reputable websites for information.

#### Support System

The availability of social services for families expecting a fetus with significant cardiac disease is part of the comprehensive care, although it is lacking in LMIC. Moreover, support groups for CHD offer psychological and information background for shared experiences. In LMICs, well-formulated support social groups are limited .

### Multidisciplinary Team (MDT)

**Fig. 2:**
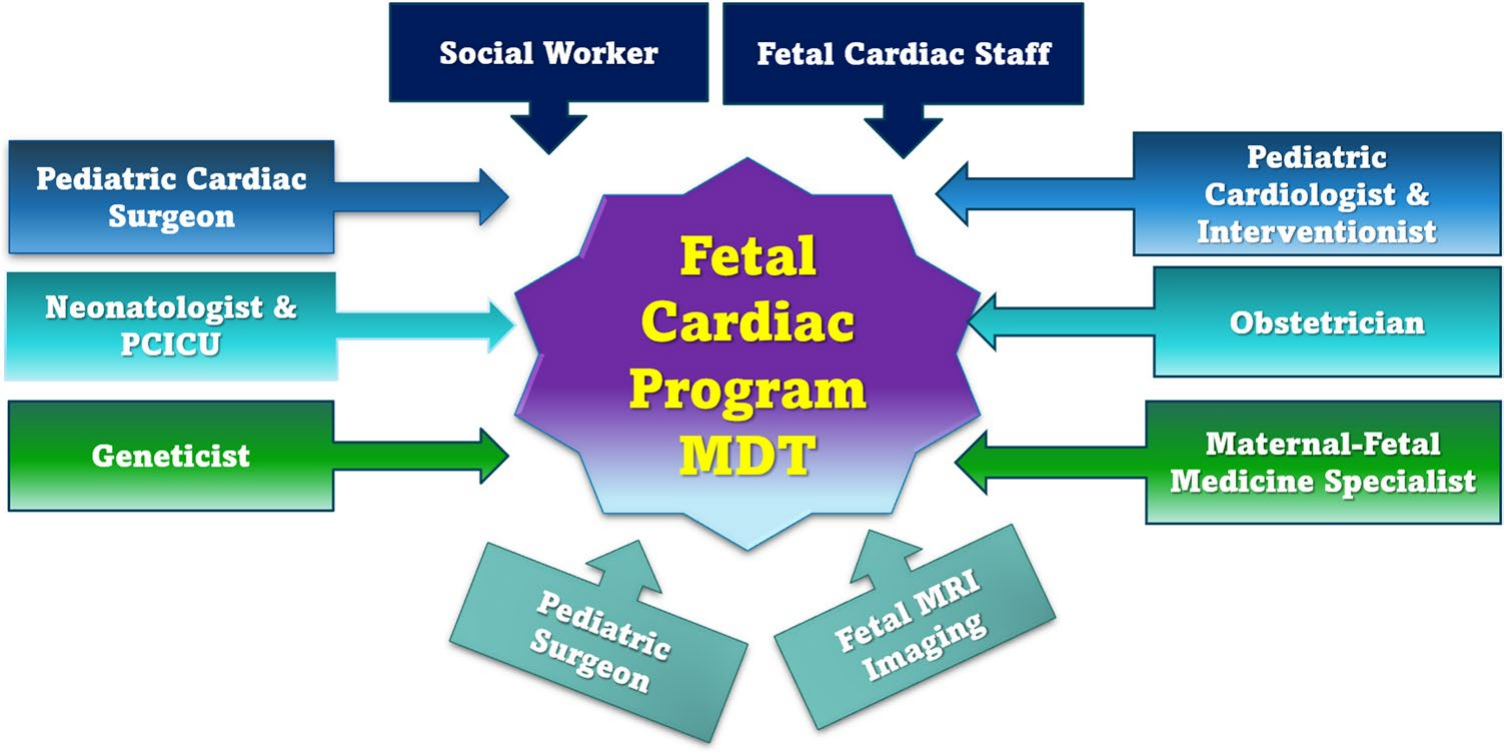
Suggested multidisciplinary team members for a fetal cardiac program

A multidisciplinary team is a mandatory collaboration to assist the fetal cardiologist in reaching a collaborative, comprehensive, customized, and individualized prenatal, natal, and postnatal care plan [20]. The assembly of experienced multispecialty panels in MDT meetings allows for better integration of services to improve the care process. A study found that prenatally diagnosed cases requiring Fontan operation were more likely to be delivered if a multidisciplinary team was involved in discussing the prognosis [26]. Team communication could be on an elective weekly/monthly basis (face-to-face meetings). However, urgent, possibly online communications allow timely decision-making for selected cases. MDT should include variable subspecialty members (see Fig. 2), such as fetal cardiologists, fetal medicine specialists, pediatric cardiologists/interventionists, pediatric cardiac surgeons, obstetricians, geneticists, and neonatologists. However, other specialties, such as pediatric surgeons and radiologists, may be needed based on other associated comorbidities. Although subspeciality could be available in tertiary centers, online consultations or videoconferencing is potential option for limited-resource services.

### Perinatal Arrangements

For cases with a confirmed diagnosis of fetal CHD, a policy should be adopted for delivery location, mode, and timing based on the fetal echocardiographic parameters, considering the severity of the cardiac defect and the expected degree of postnatal hemodynamic compromise. Risk-stratified classification of delivery room care of newborns with CHD suggested by Donofrio et al. is one of the comprehensive care plans sub-classifying the predicted instability in the delivery room as low, minimal, and high (further subclassified) [27].

Moreover, the MDT team should determine antenatally whether the delivery will be in-house or in another regional hospital or tertiary center, specifying who will be responsible for managing the admission and beds for mother and fetus, particularly in LMIC, as hospital bed availability is always a challenge for optimum care.

## Fetal Echocardiography Program Challenges in LMIC

**Fig. 3:**
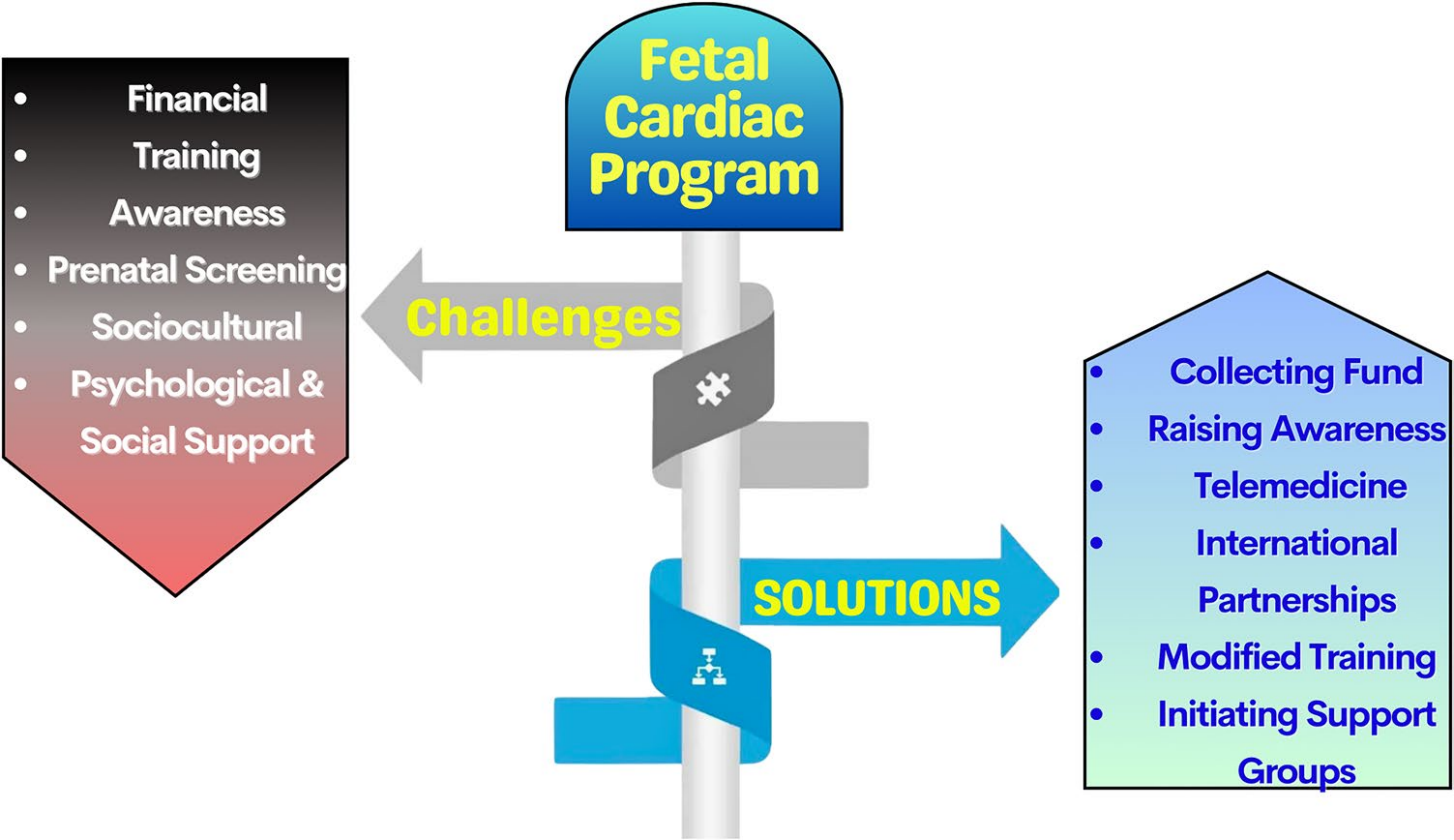
Challenges and suggested solutions for fetal cardiac program initiation

Several challenges could be a barrier to the initiation of an efficient fetal cardiac program in LMICs. In the next section, the substantial challenges are explored, followed by suggested solutions (see Fig. 3).

### Financial Requirements

Initiating a fetal cardiac program poses a high financial burden on any hospital. The limited financial funding to initiate and maintain such a service is a significant factor impacting the restriction of the service to a few centers in each country. In LMICs, there is limited expenditure on health altogether [28, 29]. Unfortunately, the limited insurance coverage in these countries leads to out-of-pocket payment for such rare services, which is considered an extrabarrier to accessing care [30].

### Staff Training Challenge

The lack of sufficiently trained pediatric cardiologists with fetal cardiology experience is a fundamental limiting factor, as a program could only be established with at least one fetal cardiologist. Another challenge is the limited accredited training programs for sonographers to help with fetal cardiac screening in LMIC [31]. Moreover, fetal cardiac sonographers are entirely unavailable in some countries.

In many LMICs, there is a shortage of health personnel providing ultrasound services [32, 33]. Moreover, ultrasound use in LMICs was not associated with reducing maternal, perinatal, or neonatal mortality [32]. In a survey study, the top barriers to ultrasound use in LMIC included, in addition to the machine, the limited access to education and training [34].

### Limited Awareness

Lack of knowledge regarding the presence and extent of fetal heart service for referring physicians such as obstetricians and for the families remains one of the main challenges in LMIC. In a survey study that included parents of children with CHD, a significant knowledge gap was proven regarding fetal echocardiography due to parent counseling inadequacy, especially in developing countries [35]. A study from India detected that awareness about the fetal echocardiography in the study population was as low as 2.2% [36]. In a study from Pakistan on causes of delayed referral to fetal echocardiography, referral personnel were found to be responsible for the delayed referral of females. Therefore, they recommended that pregnant women and gynecologists be properly educated about the importance of timely referrals for fetal echocardiography [37]. In well-developed countries, fetal cardiac referrals are an established practice among fetal medicine and obstetric teams. With high education levels, families could easily access information regarding fetal echocardiography.

### Lack of National Prenatal Obstetric Screening

The lack of national prenatal screening guidelines is one of the reasons for limited antenatal assessments, including initiating a fetal cardiac program [38]. A systematic review demonstrated that screening methods are infrequently implemented in LMICs to identify the correctable congenital anomalies due to financial, material, and human resource constraints [39]. Out of thirty-seven centers providing surgical care for CHD in 17 LMICs, only 19% of centers had universal antenatal CHD screening in their catchment area, and only 46% of these centers offered fetal echocardiography [40].

### Sociocultural Factors

There are sociocultural variabilities that impact the need to establish fetal cardiac service. In India, with the widespread availability of termination of pregnancy (TOP), 50% of fetuses with suspected CHD get TOP, indicating that the use of fetal cardiac services is suboptimal [8]. On the contrary, in Middle Eastern countries, some pregnant women refuse to peruse fetal cardiac assessment due to the unavailability of TOP due to social or religious constraints, as it is mainly prohibited except for limited indications, none of which included CHD [41].

### Psychological and Social Support

Knowing that the unborn child has a CHD carries the risk of psychological distress, anxiety, and depression for the parents. Families go through an uncertain path following a fetal diagnosis of severe CHD [42]. The challenges faced by the cardiologists caring for them overlap with those experienced by pediatric palliative care practitioners [43]. Parents and families require more information, counseling, and support than the medical team offers, even in developed societies [44–47]. This could be explained by the substantial anxiety and depression levels in parents after counseling for fetal heart disease [48]. However, there are limited organized support groups or social societies for CHDs and fetal cardiac disorders, with limited or unavailability of specialized social workers to help the families in LMICs.

## Hopes and Solutions

### Collecting Fund

Engaging governmental organizations is inevitable to sustain a specialized comprehensive medical service. It will require a budget from any hospital to maintain an efficient program with its requirements. Fetal cardiologists should advocate for initiating the program by orienting healthcare managers and stakeholders about the need for fetal cardiac service. A proposal for a grant could be a good start. Moreover, fundraising could provide a decent push to start a service, mainly for a suitable echocardiographic machine, which is one of the most expensive basic requirements. Launching fundraising activities or campaigns could be beneficial for establishing the program, such as organizing events, selling products flagging the supported program like T-shirts or pens, using online fundraising platforms, and contacting charity or nonprofit organizations.

However, for the sustainability of the service, a national commitment is required to support antenatal–perinatal cardiac care. Health policymakers should be persuaded that improving prenatal diagnosis is among the strategies to optimize the costs of newborn cardiac patients. Limited studies addressed the cost-effectiveness of prenatal detection in LMIC and subsequent perinatal management versus postnatal diagnosis. In a resource-limited setting, the cost of neonatal cardiac care was found to be significantly lower for prenatally diagnosed CHD than for postnatal diagnosis, especially for patients requiring surgery. Fetal cardiac diagnosis, compared to postnatal diagnosis, was associated with less health expenses to income ratios, which is an indicator of a financial burden on families [17]. Moreover, it is proved that prenatally detected cases have significantly lower hospitalization costs and emergency transfer costs than neonatally diagnosed cardiac cases [49, 50]. Therefore, it seems reasonable to demand a partial transfer of funds from postnatal endeavors to create and support a prenatal cardiac program.

### Raising Awareness Among Families, Physicians, and Health Policymakers

Education about fetal echocardiography should be expanded to include most of the referring physicians regarding indications, scope of service, and the value of timely referral through webinars or workshops and other scientific events. Familial awareness could be raised through parental education for families with a history of CHD. Furthermore, media and social networks could be utilized to build mass knowledge about fetal cardiac services.

Health policymakers should be aware of the cost-effectiveness of building national fetal cardiac screening programs in LMICs and referring cases that fail the screening for fetal cardiology expert evaluation and counseling [49].

### Official Telemedicine Channels

Telemedicine has been established to be valuable and practical in pediatric and fetal cardiology on large-scale services [51, 52]. Telemedicine has consistently exhibited good results in terms of health and budget. It has been widely used in the USA and Europe and has limited availability in developing and undeveloped countries [53]. Fetal tele-echocardiography could confirm diagnosis and teleconsultation could accurately determine the patient requiring referral to a tertiary center for fetal intervention, neonatal catheterization, or cardiac surgery. Telemedicine can be a potent tool to facilitate care in low- and middle-income countries. However, it has inherent challenges, such as access to the technology supporting its implementation [54].

### International Health Partnerships

Establishing international partnerships will expand knowledge and upgrade the medical service by allowing the exchange of medical expertise. It permits a better understanding of other cultures and the extent of fetal cardiac problems among nations. Therefore, it could open the door for multi-institutional, multicountry research points to gather comprehensive experiences.

### Modified Training Protocols

With the limited numbers of trained fetal cardiologists, different learning techniques could emerge and be accredited in the training process. The e-learning pediatric cardiology curriculum was tried in some developing countries with substantial success [55, 56]. Fetal cardiology could be adopted with similar rules to cover the growing need for fetal cardiologists.

Appropriate patient management relies on the ability of health personnel to use ultrasound efficiently and interpret the findings accurately. A study from the Netherlands found that despite adequate image quality, CHD was not recognized on fetal cardiac screening in 31% of cases [57]. Implementing antenatal cardiac ultrasound mass training programs can be delivered effectively with minimal impact on healthcare resources. In Wales, government-funded educational interventions allowed for ongoing training on fetal cardiac screening. The program led to an improved antenatal detection rate of CHD and a substantial decline in non-chromosomal CHD-related mortality and morbidity [58]. Qualified sonographers for fetal cardiac screening could be those with a background in midwifery or radiography. Optimal rates of prenatal detection of critical CHD can be achieved by a program for sonographer education and training [59]. Training midwives to perform fetal cardiac ultrasound screening helped expand national screening in European countries [60].

For fetal cardiac sonographers, a suggested novel ultrasound simulation application for fetal echocardiographic training is another important step toward remote learning. This could overcome the difficulties of traditional training, such as simultaneously having patients, ultrasound machines, and professors [61, 62]. Janzing et al. proved that with simulation, the students reached the same skill level as the expert group sonographers in fetal echocardiography within 6 weeks [62].

### Initiating Support Groups

Fetal cardiologists, in addition to their medical expert role, have a challenging advocacy duty in low-income settings. Advocacy should include demonstrating the family demands to the community organizations and government to help initiate support groups and availability of social services. It is helpful for parents of CHD patients to be in contact with organizations and people who can offer support and answer their questions. A CHD support group would create accessible information and materials, easy-to-navigate websites, and arrange online and in-person events, including people from different cultures [63].

## Conclusion

Initiating a successful fetal cardiac program is a substantial challenge, especially in LMIC countries, as it requires multi-aspect structural planning. Moreover, comprehensive fetal cardiac care can only be accomplished with the combined efforts of MDT and the fetal cardiologist. Several challenges could face the program initiation process; however, diverse collaborative efforts from care providers, governmental and nonprofit organizations, and the community is required for adequate building and utilization of fetal heart program resources.

## Data Availability

Not applicable.

## Abbreviations

ASE: American Society of Echocardiography
CHD: Congenital heart disease
DICOM: Digital Imaging and Communications in Medicine
LMICs: Low- and middle-income countries
MDT: Multidisciplinary team
PACS: Picture Archiving and Communication System
SDI: Socio-demographic index
TOP: Termination of pregnancy
